# An indica rice genotype showed a similar yield enhancement to that of hybrid rice under free air carbon dioxide enrichment

**DOI:** 10.1038/srep12719

**Published:** 2015-07-31

**Authors:** Chunwu Zhu, Xi Xu, Dan Wang, Jianguo Zhu, Gang Liu

**Affiliations:** 1 State Key Laboratory of Soil and Sustainable Agriculture, Institute of Soil Sciences, Chinese Academy of Sciences, East Beijing Road, Nanjing, 210008, PR China; 2 International Center for Ecology, Meteorology and Environment, School of Applied Meteorology, Nanjing University of Information Science and Technology, Nanjing 210044, China

## Abstract

Although the rice growth response to FACE (free-air CO_2_ enrichment) has been widely studied and is considered important within the scientific community, few studies have attempted to examine the effects of FACE on the yield of indica rice, which is typically the parent of indica hybrids in China. The effects of FACE on the yield, yield components, biomass, N uptake and leaf photosynthesis of Yangdao 6 Hao (an indica rice) in China were examined over 2 years. The grain yield increased over 30%, the panicle number increased 12.4% on average, and the spikelet number per panicle also showed an average increase of 8.2% at elevated CO_2_. FACE caused a significant enhancement in both the filled spikelet percentage (+5.9%) and the individual grain weight (+3.0%). Compared with three prior FACE studies on rice, a similar enhancement of yield in hybrid indica was shown under FACE, with much a higher value than for the japonica rice cultivar (approximately + 13%) because of indica’s stronger sink generation and N uptake capacity, which help coordinate the C/N balance to avoid photosynthetic acclimation. The high enhancement of the indica rice yield under FACE holds promise for improved cultivar selection for future food security.

Since 1959, the concentrations of atmospheric carbon dioxide [CO_2_] have increased from approximately 318 to 400 μmol mol^−1^ and, depending on the anthropogenic emission rates, may reach 1000 μmol mol^−1^ by the end of the century^1^. Because the photosynthetic rate in C_3_ species under the current levels of ambient [CO_2_] is still below physiological saturation levels, it is anticipated that photosynthesis, and consequently productivity, for most crops will be stimulated by the higher atmospheric [CO_2_]^2^. In theory, an increase in [CO_2_] from 380 μmol mol^−1^ to 550 μmol mol^−1^ projected for the year 2050 would increase C_3_ photosynthesis by 38%^3^, as indicated by many experimental and analytical studies^4^,^5^.

Many early FACE studies have shown that rice yield increases (9–15%) were lower than expected due to photosynthetic acclimation^5^,^6^,^7^,^8^. In addition, researchers have found marked differences among rice cultivar responses to elevated [CO_2_]^9^,^10^,^11^,^12^. Hence, active selection and breeding for high CO_2_ responsiveness among rice varieties may provide a simple and direct strategy for increasing global yields and maintaining food security with climate change, but this potential has not received sufficient attention until recently^13^. China rice-FACE studies have already revealed hybrid rice genotypes with a greater yield enhancement (above 30%) under elevated [CO_2_] than conventional rice cultivars^9^,^11^. Previous FACE studies in Japan and China previously demonstrated that japonica rice exhibits a weak response to elevated [CO_2_]^5^,^6^,^7^,^8^. Therefore, we hope to find an indica rice genotype that has a similarly high enhancement under elevated CO_2_ as that exhibited by hybrid rice and that can be used under future climatic conditions.

Yangdao 6 Hao has large panicles, a high yield potential, resistance to disease and pathogens, and an anti-lodging ability, which suggest that this cultivar may be an important gene resource for rice breeding^14^. Hence, we chose this cultivar as the study subject. The aim of this study was to investigate whether the indica rice under consideration has a similarly strong response to elevated [CO_2_] as hybrid rice using FACE (free air carbon dioxide enrichment) treatment.

## Results

### Effects of CO_2_ on grain yield

FACE significantly increased the grain yield of the rice (*P* < 0.01) (Table 1). The enhancement of the grain yield was 36.2% and 29.6% in 2012 and 2014, respectively. There was a strong effect of year on the yield response (*P* < 0.05) (Table 1), but interactions between CO_2_ and year were not detected.

### Effects of CO_2_ on yield components

As shown in Table 1, the panicle number per m^2^ was increased to a similar extent (13.5% for 2012 and 11.2% for 2014, *P* < 0.05) under elevated [CO_2_], and there was no significant interaction between CO_2_ and year. The number of spikelets per panicle increased by 9.6% for 2012 and by 6.8% for 2014, and there was a strong year effect (*P* < 0.01). There was no interactive effect of CO_2_ × year on spikelets per panicle (Table 1).

For the filled spikelet percentage, FACE rice showed a similar increase in the two years (5.8% for 2012 and 5.9% for 2014). The individual grain weight increased by 3.5% and 2.3% for FACE vs. ambient plants in 2012 and 2014, respectively (Table 1). The interaction between CO_2_ and year was not detected for the two yield components.

### Effects of CO_2_ on phenology, shoot and tiller biomass, and plant height

**Figure 1:**
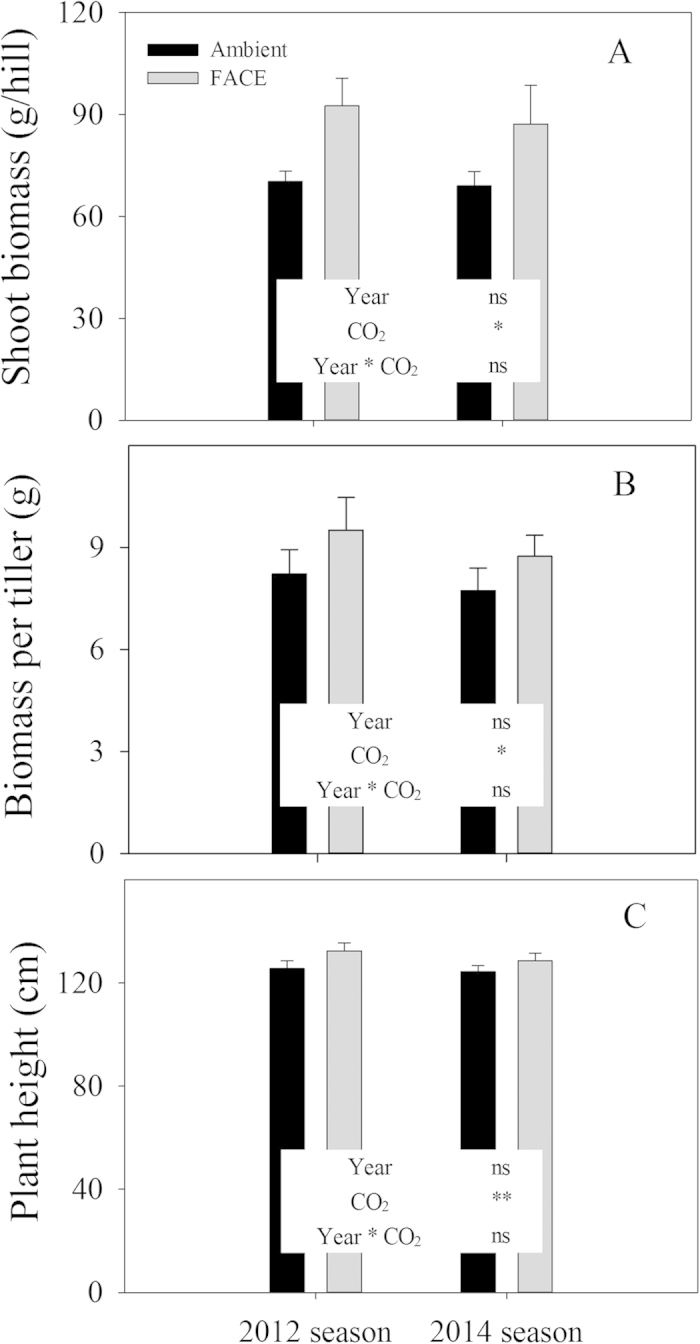
Shoot biomass and plant height of plants grown under ambient CO_2_ and free-air CO_2_ enrichment conditions (FACE, 200 μmol mol^−1^ above ambient conditions) at maturity in the 2012 and 2014 seasons. *Indicates *P* < 0.05 between CO_2_ levels. ns, not significant; *P < 0.05; **P < 0.01.

There was no change in phenology upon reaching 50% panicle emergence and grain maturity (Table 2). In contrast, FACE significantly increased the shoot and tiller biomass and plant height at maturity. When averaged across years, the shoot and tiller biomass and plant height were increased by 29.0%, 14.3% and 4.5%, respectively. There was no interactive effect of CO_2_ × year on phenology, shoot and tiller biomass or plant height (Fig. 1 and Table 2).

### Effects of CO_2_ on gas exchange

**Figure 2:**
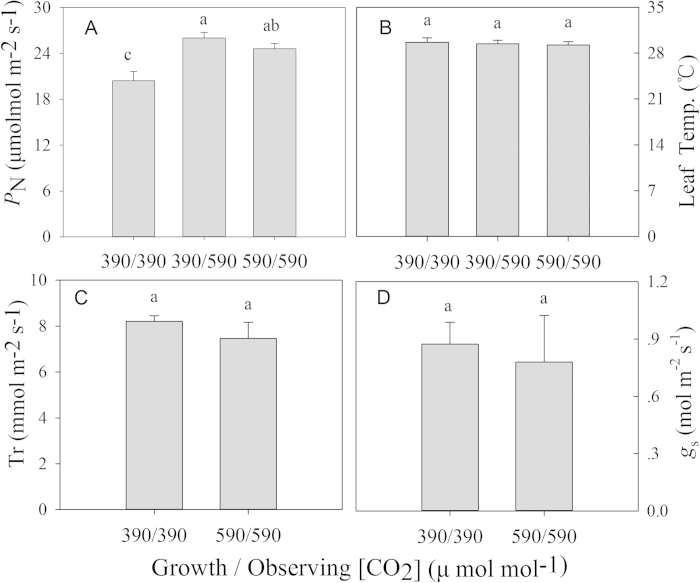
Photosynthetic gas exchange measurements taken at the respective growth [CO_2_] conditions (ambient and FACE: 390 and 590 μmol mol^−1^, respectively) on a light-saturated flag leaf at the mid-filling stage in the 2014 season. (**A**) Net photosynthetic carbon assimilation rate (*P*_N_); (**B**) leaf temperature; (**C**) transpiration rate (TR); (**D**) stomatal conductance to water vapor (*g*_s_). 390/390 and 390/590: grown under ambient conditions and measured at 390 and 590 μmol mol^−1^ [CO_2_], respectively; 590/590: grown under FACE conditions and measured at 590 μmol mol^−1^ [CO_2_]. The mean was the average of 3 replications (n = 3) ± SD. ns, not significant; *P < 0.05; **P < 0.01.

There was a significant stimulation of flag leaf photosynthesis with elevated [CO_2_] (relative to ambient) observed at the mid-filling stage for plants grown under either ambient or FACE conditions (Fig. 2A). There was no difference in the net photosynthetic carbon assimilation rate of plants grown under ambient and FACE conditions when measured at the same [CO_2_] (590 μmol mol^−1^), indicating that the photosynthetic efficiency was not reduced by elevated [CO_2_], even at the mid-filling stage (Fig. 2A). The leaf temperatures were not different during measurement. The stomatal conductance and transpiration were not significantly different under ambient CO_2_ and FACE conditions, although there was a declining trend due to the elevated [CO_2_] (Fig. 2B,C). Considering the meaningless in stomatal conductance and transpiration with elevated [CO_2_] for plants grown under ambient conditions, we did not show these parameters.

### N uptake

**Figure 3:**
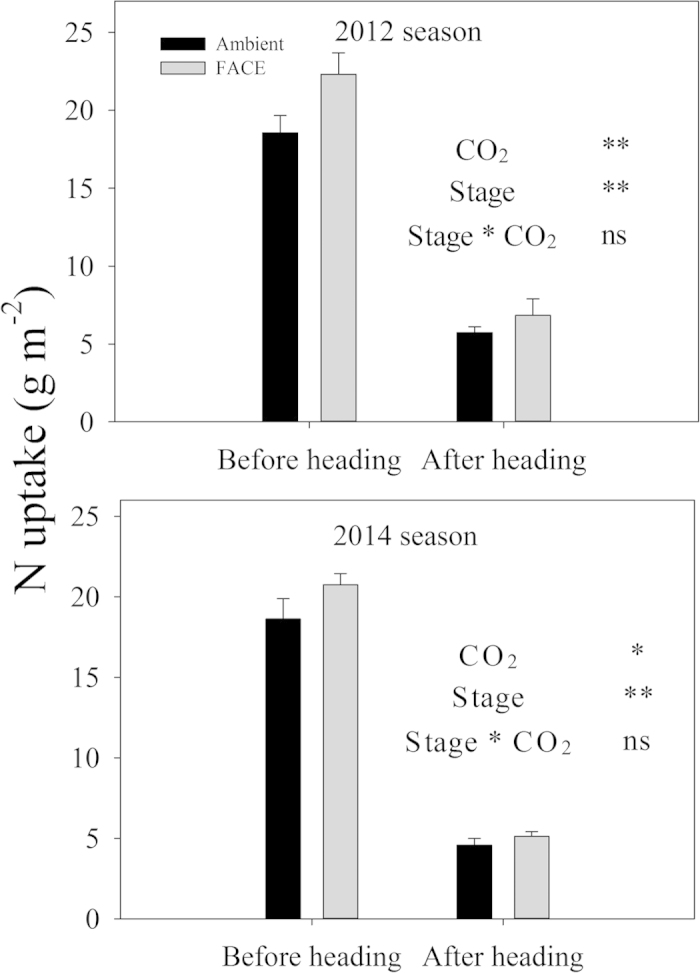
Shoot N uptake during the vegetative (from transplanting to heading) and reproductive (from heading to maturity) stages of rice grown under ambient CO_2_ and free-air CO_2_ enrichment (FACE, 200 μmol mol^−1^ above ambient) conditions in the 2012 and 2014 seasons. ns, not significant; *P < 0.05; **P < 0.01.

Averaged across two years, the N uptake in the FACE condition was increased by 15.9% during the vegetative stages (from transplanting to heading) and 15.7% during the reproductive stages (from heading to maturity) (Fig. 3). There was a clear variation between the two growth periods (Fig. 3), but the CO_2_ effect and stage interactions were not significant.

## Discussion

The 2-year FACE study showed that elevated [CO_2_] increased the yield of Yangdao 6 Hao by over 30% (Table 1), which was a similar enhancement to that of hybrid rice in previous reports of China FACE^9^,^11^. This value is far higher than the range reported in inbred japonica rice FACE studies^5^,^8^,^10^,^12^. Undoubtedly, this study reveals the potential for taking full advantage of higher [CO_2_] levels for inbred rice varieties.

The FACE condition significantly enhanced the panicle density by 12.4% in Yangdao 6 Hao (Table 2), 10.3% in Shanyou 63 and 7.8% in Liangyoupeijiu^9^,^11^, all of which are smaller than the 18.8% increase in Wuxiangjing 14 in China Wuxi FACE^8^. The different responses to FACE among these varieties may be associated with differences in the dimensions of the leaf laminae^9^. Compared to rice cultivars with small and erect leaf blades (e.g., Wuxiangjing 14), Yangdao 6 Hao, Shanyou 63 and Liangyoupeijiu, with large and drooping leaves, would suffer more from mutual shading during crop development, thus presumably resulting in a weak stimulation of CO_2_ induction for tillering and the resulting panicle number^9^.

In the present study, the number of spikelets per panicle in Yangdao 6 Hao increased by 8.2%, which was slightly lower than in hybrid indica but higher than in japonica under FACE conditions (Table 2). Shimono *et al*. reported that the N uptake before the heading stage was closely correlated with the spikelet density rather than the [CO_2_] and the cultivar type^10^. N uptake by Yangdao 6 Hao increased by 15.9% before the heading stage under elevated [CO_2_]. The enhanced N uptake before heading was beneficial to increasing the spikelet number for Yangdao 6 Hao under FACE (Tables 1 and 2). In addition, the substantial enhancement in panicle size in this study was supported by the corresponding responses of plant height (Fig. 1A) and shoot biomass (Fig. 1B, Table 2) to elevated CO_2_, which were consistent with the findings that the height and tiller biomass were correlated positively and significantly with panicle size^15^. The spikelet number per panicle is the result of the difference in the number of differentiated and degenerated spikelets^8^. It is well accepted that cytokinins are mainly produced in the plant root and distributed in the shoot by the transpiration stream^16^,^17^, which impacts rice spikelet formation and development. The decreased spikelet number of japonica rice varieties with a low response to elevated CO_2_ in China FACE (Table 2) may be attributed to the decrease in root activity that reduces cytokinin synthesis^18^,^19^. This condition is unlikely to be the case for Yangdao 6 Hao, in which the spikelet number per panicle and the N uptake of the vegetative and reproductive stages were significantly increased by the elevated CO_2_. These findings suggest that Yangdao 6 Hao can maintain root activity for cytokinin synthesis under elevated CO_2_. However, the potential physiological and molecular mechanisms underlying the different responses to elevated CO_2_ require further study.

Elevated [CO_2_] increased the actual grain sink per panicle (filled spikelet ratio × spikelet number × per panicle grain weight) by 18.0% in this study (Table 2), which is slightly lower than in hybrid rice but higher than the values obtained in previous FACE studies^6^,^8^,^9^. Obviously, grain-filling abilities are related to photosynthate assimilation after heading. At the mid-filling stage, Yangdao 6 Hao maintained a strong increase in the net photosynthetic carbon assimilation rate and avoided photosynthetic acclimation under elevated [CO_2_] (Fig. 2A). This response is similar to that exhibited by the hybrid rice Shanyou 63, for which elevated [CO_2_] resulted in an increased spikelet number and grain weight, increased sink:source ratio, and continued stimulation of photosynthesis up to grain maturity^20^. Overall, these results suggest that the greater response of this rice line to elevated [CO_2_] may be associated with enhanced panicle sinks relative to sources and the ability to maintain photosynthetic capacity during grain development. Under FACE conditions, these rice lines can avoid the photosynthetic acclimation that is common in C_3_ cereals^5^,^12^,^20^,^21^. As a result of the balance between carbon and nitrogen metabolism within the leaf under elevated [CO_2_], the greater sink and the significant enhancement of N uptake during the filling stage (Fig. 2), these plants avoided the suppression of photosynthetic system genes and the resulting decrease in photosynthetic capacity^20^,^21^. Maintaining photosynthetic efficiency during the grain-filling stage ensures a strong yield enhancement under FACE conditions.

This is the first study to confirm that an inbred indica genotype exhibits a yield enhancement similar to that of hybrid rice under elevated [CO_2_]. To ensure food security in the future, additional indica genotypes with potentially strong responses to elevated [CO_2_] should be evaluated to take full advantage of the predicted increases in [CO_2_].

## Materials and Methods

### Research site

The experiment was conducted at the FACE facility located in Zhongcun Village (119°42’0”E, 32°35’5”N), Yangzhou City, Jiangsu Province, a typical Chinese rice-growing region^22^. The soil was classified as Shajiang-Aquic Cambiosol with a sandy loam texture. The soil properties at 0–15 cm relevant to this experiment are as follows: bulk density 1.16 g cm^−3^, soil organic carbon 18.4 g kg^−1^, total nitrogen 1.45 g kg^−1^, available phosphorous 10.1 mg kg^−1^, available potassium 70.5 mg kg^−1^, and pH 6.8^23^.

The operation and control systems for the FACE facilities were the same as those used at the Japan FACE site^24^: three identical octagonal rings with the target [CO_2_] concentration at the center 200 μmol mol^−1^ higher than ambient conditions (hereinafter referred to as FACE) and three comparison rings of ambient [CO_2_]. During the 2012 and 2014 seasons, the average daytime [CO_2_] levels at the canopy height during the experiment were 378 and 577 μmol mol^−1^ and 394 and 590 μmol mol^−1^ for the ambient and elevated FACE rings, respectively. The average temperature during the growing season was 24.4 °C and 22.1 °C for 2012 and 2014, respectively.

### Rice cultivation

An indica rice (*Oryza sativa*) line (Yangdao 6 Hao) was selected in this study. Seeds were sown at ambient [CO_2_] on May 20, 2012 and 2014. The seedlings were then manually transplanted to ambient and elevated FACE rings at a density of one seedling per hill on June 21. The spacing of the hills was 16.7 cm  ×  25 cm (equivalent to 24 hills m^−2^). N was applied as a basal dressing (40% of the total) 1 day prior to transplanting and as a top dressing at early tillering (30% of the total) and at the panicle initiation (PI) stage (30% of the total) at 22.5 g N m^−2^. Phosphorous (P) and potassium (K) were applied as a compound fertilizer at 9 g P_2_O_5_ m^−2^ and 9 g K_2_O m^−2^; both P and K were applied as a basal dressing 1 day before transplanting.

### Photosynthetic gas exchange measurement

Before the measurements, we measured the Chl content in 5–6 flag leaves per treatment plot non-destructively using a Chl meter (SPAD-502, Konica Minolta Optics, Inc., Japan). We then used two leaves with representative Chl content for the gas exchange measurements with a portable photosynthesis system with blue and red LED light sources (LI-6400, LI-COR Bioscience, USA). This measurement was conducted between 09:30 and 14:30 h on September 21, 2014 (mid-filling stage); the block temperature in the cuvette was fixed at 28 °C, and the photosynthetic photon flux density was fixed at 1,800 μmol m^−2^ s^−1^, with a flow rate of 500 μmol s^−1^. A 6400-01 CO_2_ injector attached to the main system was used to control the [CO_2_] in the cuvette. *P*_N_ at the respective [CO_2_] (390 and 590 μmol mol^−1^ for the control plants and 590 μmol mol^−1^ for the FACE plants) was recorded after the stomatal conductance had stabilized.

### Rice sampling and biomass measurements

The rice plants were sampled at the heading stage and at grain maturity. Six hills per plot were randomly selected and destructively sampled. The samples were separated into green and senescent leaves, stems (including leaf sheaths), and panicles.

All the plant parts were oven-dried at 80 °C to constant weight before being weighed. The N content in plant tissue was measured using an elemental analyzer (PE 2400, Series II CHNS/O, US). The N uptake was determined by multiplying the N content by the biomass of the panicles, stems and leaves.

The grain yield and yield components were measured according to Zhu *et al*.^20^. At the maturity stage, the grain yield component characteristics (i.e., panicles per m^2^, spikelet number per panicle, filled spikelet percentage and individual grain weight) were tested using six hills of rice. In addition, a 1.5 m^2^ area of rice was harvested at ground level and separated into straw and grain components. The collected grains (seeds) were soaked in 1.00 specific gravity tap water, and the number of sunken and floated grains were counted to determine the filled spikelet percentage. The dry weight of the ripened (sunken) grains was measured after they were oven dried at 80 °C for 72 h. The weight per grain and grain yield were expressed by incorporating a 14% moisture content basis^8^.

### Statistical analysis

The experimental design was a split plot arranged within a randomized complete block with 3 replications (three rectangular paddy fields). Using the software Statistical Package for the Social Sciences 19.0 (SPSS Inc., Chicago, USA), we first performed an analysis of variance for the main factors of [CO_2_] and year on the yield and its components, including biomass and plant height, as shown in Table 1 and Fig. 1. We also performed an analysis of variance for the main factors of [CO_2_] and stage on N uptake, illustrated in Fig. 3. In addition, post hoc comparisons were performed to detect the effects of [CO_2_] on the gas-exchange parameters, as shown in Fig. 2.

## Additional Information

**How to cite this article**: Zhu, C. *et al*. An indica rice genotype showed a similar yield enhancement to that of hybrid rice under free air carbon dioxide enrichment. *Sci. Rep*. **5**, 12719; doi: 10.1038/srep12719 (2015).

## Figures and Tables

**Table 1:** Effect of free air CO_2_ enrichment (FACE) on yield and its components of Yangdao 6 Hao in the 2012 and 2014 seasons.

Year	CO_2_	Panicle number (m^−2^)	Spikelets per panicle	Filled ratio (%)	Single grain weight (mg)	Yield (g m^2^)
2012	Ambient	206.2 ± 9.6	156 ± 8.6	79.2 ± 0.4	34.7 ± 0.5	882.7 ± 40.0
	FACE	234.0 ± 13.1	170.9 ± 8.7	83.8 ± 1.2	35.9 ± 0.2	1201.8 ± 83.6
	Change %	13.5	9.6	5.8	3.5	36.2
2014	Ambient	215.1 ± 17.1	142.1 ± 7.2	76.4 ± 3.1	34.4 ± 0.4	798.9 ± 66.0
	FACE	239.3 ± 18.3	151.7 ± 3.2	80.9 ± 1.9	35.2 ± 0.3	1035.0 ± 118.1
	Change %	11.2	6.8	5.9	2.3	29.6
ANOVA results						
Year	ns	**	*	*	*	
CO_2_	*	*	**	**	**	
Year* CO_2_	ns	ns	ns	ns	ns	

ns, not significant; *P < 0.05; **P < 0.01.

**Table 2:** Summary of experimental background (years, locations, test cultivars), absolute response (d) in phenology and relative responses (%) in plant height, grain yield and its components of rice crops grown under elevated [CO_2_] (ambient + 200 μmol mol^−1^) relative to ambient [CO_2_] in the Japanese and Chinese rice FACE experiments.

Item	Japan FACE	Japan FACE	China FACE	China FACE		China FACE
Experimental location	Iwate (39°38′N, 140°57′E)	Tsukubamirai (35°58′N, 139°60′E)	Wuxi (31°37′N, 120°28′E)	Yangzhou (32°35.5′N, 119°42′E)		Yangzhou (32°35.5′N, 119°42′E)
Test variety	Akitatakomachi	Koshihikari	Wuxiangjing 14	Shanyou 63	Liangyoupeijiu	Yangdao 6 Hao
Genotype	Japonica	Japonica	Japonica	Hybrid indica	Hybrid indica	Indica
Experimental years	1998–2000	2010–2012	2001–2003	2004–2006	2005–2006	2012 and 2014
N level (g m^−2^)	4, 8 or 9, 12 or 14	8, 12	12, 25, 35	12.5, 25	12.5, 25	22.5
[CO_2_] target	+200 ppm	+200 ppm	+200 ppm	+200 ppm	+200 ppm	+200 ppm
Phenology						
Heading	−2 d	−2 d	−3.4 d*	+1.3 d ns	−1.5 d*	0 d ns
Maturity	−1–0 d	–	−5.8 d*	0 d ns	−0.1 d ns	0 d ns
Plant height	–	–	−2.2% ns	+6.7%*	+6.1%*	+4.5%*
Yield	+12.8%**	+16.3%*	+12.8%*	+34.1%**	+30.1%**	32.9%**
Yield components						
Panicle number per m^2^	+8.6%*	+9.3%**	18.8%**	+10.3%**	+7.8%**	+12.4%*
Spikelet number per panicle	+1.9%**	+2.2% ns	−7.6%**	+10.3%**	+9.6%**	+8.2%*
Filled spikelet percentage	+0.8% ns	+3.4%*	+4.9%**	+4.9%**	+5.3%**	+5.9%**
Single grain weight	+1.3%*	+0.5% ns	+1.3%**	+4.3%**	+4.4%**	+3.0%**
Data sources	Kobayashi *et al*.^24^, Kim *et al*.^6^	Hasegawa *et al*.^12^	Yang *et al*.^8^	Liu *et al*.^9^	Yang *et al*.^11^	Table 2 and Fig. 1 in this paper
