# Corrigendum: An indica rice genotype showed a similar yield enhancement to that of hybrid rice under free air carbon dioxide enrichment

**DOI:** 10.1038/srep15312

**Published:** 2015-10-21

**Authors:** Chunwu Zhu, Xi Xu, Dan Wang, Jianguo Zhu, Gang Liu

Scientific Reports 5: Article number 12719; 10.1038/srep12719 published online: 06242015; updated: 10212015.

In the original version of this Article, Chunwu Zhu and Jianguo Zhu were incorrectly listed as being affiliated with ‘International Center for Ecology, Meteorology and Environment, School of Applied Meteorology, Nanjing University of Information Science and Technology, Nanjing 210044, China’. In addition, Chunwu Zhu and Jianguo Zhu should only be affiliated with ‘State Key Laboratory of Soil and Sustainable Agriculture, Institute of Soil Sciences, Chinese Academy of Sciences, East Beijing Road, Nanjing, 210008, PR China’. These errors have now been corrected in both the HTML and PDF versions of the Article.

